# Circumcision for the Relief of Acne

**Published:** 1902-02-15

**Authors:** 


### CIRCUMCISION FOR THE RELIEF OF ACNE.

A curious case is reported by Dr. Ussery,[1] of Kentucky, in which a case of aggravated acne of the face, of five years' standing, got well rapidly after the performance of circumcision. The prepuce was not long, and there were no adhesions. Before the wound healed, there was an improvement in the acne, and at the end of three months the last pustule had disappeared.

[1] New York Med. Rec., Feb. 1.

